# Oxidized cell-free DNA as a stress-signaling factor activating the chronic inflammatory process in patients with autism spectrum disorders

**DOI:** 10.1186/s12974-020-01881-7

**Published:** 2020-07-16

**Authors:** Galina V. Shmarina, Elizaveta S. Ershova, Natalia V. Simashkova, Svetlana G. Nikitina, Julia M. Chudakova, Natalia N. Veiko, Lev N. Porokhovnik, Anna Y. Basova, Antonina F. Shaposhnikova, Daria A. Pukhalskaya, Vladimir M. Pisarev, Natalia J. Korovina, Natalia L. Gorbachevskaya, Olga A. Dolgikh, Marina Bogush, Sergey I. Kutsev, Svetlana V. Kostyuk

**Affiliations:** 1 Research Centre for Medical Genetics, Moscow, Russia; 2 I.M. Sechenov First Moscow State Medical University (Sechenov University), Moscow, Russia; 3 G.N. Gabrichevsky Institute of Epidemiology and Microbiology, Moscow, Russia; 4 Scientific Center for Mental Health, Moscow, Russia; 5 G.E. Sukhareva Scientific-Practical Centre for Mental Health of Children and Adolescents, Moscow, Russia; 6 V. A. Negovsky Research Institute of General Reanimatology, Federal Research and Clinical Center of Intensive Care Medicine and Rehabilitology, Moscow, Russia; 7 Rowan University Biological Sciences Department, Science Hall, Glassboro, NJ USA

**Keywords:** Autism spectrum disorders, Cell-free DNA, NFкB1, IL-1beta, IL-8, IL-17A, Oxidized DNA fragments

## Abstract

**Background:**

Autism spectrum disorders (ASD) are known to be associated with an inflammatory process related to immune system dysfunction. This study’s aim was to investigate the role of cell-free DNA in chronic inflammatory process in ASD patients.

**Methods:**

The study included 133 ASD patients and 27 healthy controls. Sixty-two ASD patients were demonstrated to have mild-to-moderate disease severity (group I) and 71 individuals to have severe ASD (group II). Plasma cell-free (cf) DNA characteristics, plasma cytokine concentrations, expression of the genes for *NFкB1* transcription factor and pro-inflammatory cytokines *TNFα*, *IL-1β* and *IL-8* in peripheral blood lymphocytes (PBL) of ASD patients, and unaffected controls were investigated. Additionally, in vitro experiments with oxidized DNA supplementation to PBL cultures derived from ASD patients and healthy controls were performed.

**Results:**

The data indicates that ASD patients have demonstrated increased cfDNA concentration in their circulation. cfDNA of patients with severe ASD has been characterized by a high abundance of oxidative modification. Furthermore, ASD patients of both groups have shown elevated plasma cytokine (IL-1β, IL-8, IL-17A) levels and heightened expression of genes for *NFкB1* nuclear factor and pro-inflammatory cytokines *TNFα*, *IL-1β*, and *IL-8* in PBL. In vitro experiments have shown that NF-κB/cytokine mRNA expression profiles of ASD patient PBL treated with oxidized DNA fragments were significantly different from those of healthy controls.

**Conclusions:**

It may be proposed that oxidized cfDNA plays a role of stress-signaling factor activating the chronic inflammatory process in patients with ASD.

## Introduction

Autism spectrum disorders (ASD) are characterized by social communication deficits, language impairments, and stereotypical patterns of behavior, interest, or activity [1]. ASD manifest in early childhood and are followed by clinical disturbances of development [2]. In recent years, ASD are associated with an inflammatory process related to immune system dysfunction [1, 3–5]. An increase in pro-inflammatory cytokines is often considered as the hallmark of the inflammatory process in ASD [6–14]. Elevated pro-inflammatory cytokines in ASD are linked to the behavioral stereotypies and disease progression [6]. Exaggerated cytokine synthesis was found in vitro in experiments on a culture of stimulated peripheral blood monocytes derived from children with ASD [15]. Pro-inflammatory mediators and immunoactive complexes can reach higher brain centers from the blood stream and provoke neuroinflammatory processes [16]. Analysis of postmortem brain samples of ASD patients, as well as animal autism model experiments, showed signs of the inflammatory response in the central nervous system (CNS) [17–19].

Several hypotheses were suggested to explain the etiology of inflammatory process in ASD. One of them postulates a link between dysregulation of maternal immune system during pregnancy and exaggerated titers of autoantibodies that are transmitted from the mother to the fetus [20–22]. Another possible cause could be dysbacteriosis and abnormal gut permeability for bacterial invasion [23, 24]. However, the etiology of inflammatory process in ASD patients still requires unambiguous clarification. The search for inducers that determine the long-term maintenance of an elevated level of pro-inflammatory cytokines in the patient’s body remains the topical issue of child psychiatry.

As shown recently, the immune system can be activated not only by foreign environmental stimuli such as bacteria and viruses, but also by endogenous biomolecules termed damage-associated molecular patterns (DAMPs) [25]. The DAMPs are endogenous molecules that are released from damaged or dying cells and activate innate immunity via interaction with pattern recognition receptors (PRR). Though DAMPs activate the host defenses, they can contribute to the pathologic inflammatory response. Most well-studied DAMPs are proteins (HMGB1, S100, heat shock proteins (HSP)), lipoproteins, and products of their oxidation [25–28]. Until recently circulating DNA released from dying cells was not considered a biologically active molecule of the DAMP pool, as there was experimental data showing that genomic mammalian DNA has virtually no immunostimulating effect.

DNA fragments that circulate outside cells in the intercellular fluid or in cell culture medium are termed “cell-free DNA” (cfDNA) [29]. There are several hypotheses on the origin of cfDNA. The two fundamental hypotheses formation of the pool of extracellular nucleic acids as a result of cell death (“cell death” hypothesis) and active releasing DNA by living cells (“metabolic DNA” hypothesis) [30–32]. The interest in cfDNA revolves around the possibility to use cfDNA as a marker for diagnostics. In cfDNA samples isolated from peripheral blood plasma or serum, tumor DNA can be found in cancer or fetal DNA during pregnancy. This allows performing genomic analysis of the tumor or fetus without resorting to biopsy. Determining the level of cfDNA allows, in some instances, the estimation of cell death rate in pathology or damaging factor exposure [33–35]. Additionally, cfDNA circulates in the blood stream and may affect the cell’s functional activity.

Recently, a potential role of oxidative stress in pathogenesis of psychoneurological disorders such as in autism has come under investigation [36, 37]. Pre-clinical and clinical data suggest that the mental disorders are characterized by a higher level of oxidation biomarkers and a lower level of antioxidant defense biomarkers in the brain and peripheral blood [37]. Under oxidative stress conditions, the degree of oxidative modification of cellular DNA and rate of cell death are elevated. Dying cells release damaged DNA, thus introducing oxidized DNA fragments in the cfDNA pool [38, 39]. Oxidized cellular DNA can be a stress-signaling molecule, which activates the transcription factor NF-kB, resulting in the synthesis of pro-inflammatory cytokines and induction of inflammation [40–42]. Thus, cfDNA may be a link between the progression of oxidative stress and induction of the inflammatory process in ASD patients.

Investigation of biological activity of oxidized DNA fragments towards human cells is important not only for the development of novel approaches to disease diagnostics, but also for understanding mechanisms that underlie the progression of pathologic process. Here we analyzed cfDNA parameters of ASD children and healthy controls of the same age as well as studied the role for oxidized DNA fragments in immune system activation and inflammatory response development.

## Methods

### Patients and healthy controls

There were 133 patients with ASD (102 males and 31 females, 4–12 years old) who had attended the G.E. Sukhareva Scientific-Practical Centre for Mental Health of Children and Adolescents and/or the Mental Health Research Center in Moscow between February 2016 and December 2018 enrolled in the study. ASD was diagnosed using the criteria established by the DSM-5 [2]. Additionally, the patients underwent the following tests:

1. Autism Mental Status Examination (AMSE) [43] is an 8-item observational assessment that prompts the examiner to observe and document patients’ social, communicative, and behavioral functioning in the context of a routine clinical examination. The AMSE was developed by psychiatrists with autism expertise and is intended to guide clinical judgment in the context of diagnostic decision-making [44].
2. Childhood Autism Rating Scale (CARS) [45] is a scale for the quantification of the severity of autism pathology. The CARS assesses the child on a scale from 1 to 4 in each of 15 dimensions or symptoms (relating to people; emotional response; imitation; body use; object use; listening response; fear or nervousness; verbal communication; non-verbal communication; activity level; level and reliability of intellectual response; adaptation to change; visual response; taste, smell and touch response; and general impressions). Total scores of or above 30 strongly suggest the presence of autism. Children who have a score from 30 to 36 have mild to moderate autism, while those with scores ranging from 37 to 60 points have severe autism [46, 47].
3. Communication Questionnaire (SCQ) is a parent questionnaire designed for detecting risk for ASD [48]. The SCQ was originally designed as a screening tool for children 4 years of age or older enrolled in epidemiological research or for studies comparing individuals with ASD and other clinical groups [49].

Exclusion criteria included (1) neurodevelopmental disorders of known etiology (Rett syndrome, fragile X syndrome, or tuberous sclerosis, etc.); (2) clinically significant sensory or motor impairment; (3) significant medical conditions known to affect brain development (neonatal brain damage, genetic and/or metabolic syndromes involving the CNS, severe nutritional or psychological deprivation); (4) and a history of inflammatory disorders and allergies.

As in the other studies [47, 50–53], the patients were divided into two groups based on their CARS scores. Group I (*n* = 62) consisted of patients with mild-to-moderate disease severity (the CARS scores of 30–36). Group II (*n* = 71) included patients with severe ASD (CARS scores more than 36).

A control group included 27 healthy children matched to an ASD group by gender and age. For each subject of the control group, a physical examination, visual analysis of expert-level EEG and comparative EEG-mapping of the brain, and a blood analysis were performed in order to exclude any subclinical condition. None of the controls belonged to the same families as the ASD cases.

The parents of each child signed an informed consent for venous blood sampling (8 mL) and conducting research with the biomaterial sampled from the child.

The design of the study was approved by the Ethics Committee of Research Centre for Medical Genetics.

### Blood collection and blood cell population assessment

Blood was collected in EDTA and Li-Heparin tubes by venipuncture. The tubes were centrifuged at 400×*g* for 10 min at 4 °C to pellet the cells. Plasma was collected, aliquoted, and stored at − 70 °C for up to 3 months. ASD patients and healthy controls who had plasma samples with signs of hemolysis were excluded from the study. Major blood cell populations (WBC, neutrophils, lymphocytes, monocytes, eosinophils, platelets) were assessed in the fresh blood samples using the hemocytometer Abacus 5 “Diatron”. There were no significant differences between ASD children and healthy controls.

### Plasma cfDNA concentration

Cells were removed from the EDTA blood samples by centrifugation at 400×*g*, followed by mixing 3 mL of plasma with 0.3 mL of the solution containing 1% sodium lauryl sarcosylate, 0.02 M EDTA, and 75 μg/mL RNAse A (Sigma, USA). This was incubated for 45 min, then treated with proteinase K (200 μg/mL, Promega, USA) for 24 h at 37 °C. After two cycles of the purification with saturated phenolic solution, DNA fragments were precipitated by adding two volumes of ethanol in the presence of 2 M ammonium acetate. The precipitate was then washed with 75% ethanol twice, then dried and dissolved in water. The concentration of DNA (cfDNA index) was determined by measuring fluorescence intensity on «LS 55» («PerkinElmer», England) spectrometer after DNA staining with the PicoGreen (Molecular Probes/Invitrogen, CA, USA). Relative standard error of the index cfDNA was 10 ± 4%.

### Plasma nuclease activity

Nuclease activity levels in Li-Heparin plasma samples were measured by the radial diffusion technique based on radial enzyme diffusion in an agarose gel containing the substrate (dsDNA). Plasma samples (2 μl) were added to radial wells (1-mm diameter) in 1% agarose gel containing 0.05 М Tris рН 7.6, 10 mM magnesium chloride, 1 mM calcium chloride, 25 μg/mL ethidium bromide, and 0.5 mg/mL chicken DNA and were incubated at 37 °C overnight in a wet chamber. The gel was visualized in ultraviolet light on the Gel Doc XR system (BioRad, USA). Enzymatic activity was calculated from a standard curve constructed from the studies on the hydrolysis of chicken DNA by bovine pancreatic DNase (SigmaAldrich) of known concentrations.

This assay can determine a range of 1 fg to 1 pg of DNase in 1 mL serum samples within 30 min. One unit of enzyme assayed corresponds to 1 ng of purified human DNase I. The relative standard error of the index DNase 1 was 15 ± 5%.

### Determining the rate of oxidation marker 8-oxo-7,8-dihydro-2′-deoxyguanosine (8-oxodG) in cfDNA samples

Membrane (Extra C) was moistened with 20хSSC solution and dried. The cfDNA (10 ng/mL) solution in ТЕ buffer were applied to the prepared filter in an amount of 2 μL per dot. From each sample, 3–5 dots were applied. Standard samples of oxidized genomic DNA with known content of 8-oxodG were applied to the same filter in order to plot a calibration curve of signal intensity depending on the content of 8-oxodG in the sample. Standard samples of human oxidized genomic DNA were obtained via reaction of DNA with hydrogen peroxide as previously described [54]. The content of 8-oxodG in the control samples was determined by the ESI-MS/MS method using a AB SCIEX 3200 Qtrap machine. The filter was heated at 80 °С under vacuum for 30 min. For carrying out 8-oxodG detection, special equipment was used: an oven for hybridization, which enables temperature adjustment and has a swinging platen for mixing the solution above the membrane. The membrane was blocked (30 min, 30 °С) in a solution of 0.1% fat-free milk, 0.1% gelatin, Tris-HCL buffer, pH 7.5, 0.1 M sodium chloride. The membrane filter was then treated for 30 min (25 °С) with a conjugate of biotin with antibody to 8-oxodG (1 μg/mL) in solution A (0.1 M Tris-HCL buffer, pH 7.5, 0.1 M sodium chloride). The filter was washed (3 × 10 min) with solution A. Then, the membrane filter was treated for 20 min (25 °С) with a conjugate of streptavidin with alkaline phosphatase (1 μg/mL, Sigma) in solution B (0.1 M Tris-HCL buffer, pH 7.5, 0.1 M sodium chloride, 0.005 M magnesium chloride). The filter was washed (3 × 10 min) with solution B. Then, the filter was placed in a solution of substrates for alkaline phosphatase (Tris-HCL buffer, pH 9.5, 0.1 M sodium chloride, 0.005 M magnesium chloride, 4.4 μL/mL NBT and 3.3 μL/mL BCIP). The reaction was conducted in a dark room at 37 °С under visual control of emerging stained violet dots. After the reaction finished, the filter was washed with water and dried in the dark. The dried filter was then scanned. For the quantitative analysis of the dots, a special software was used, namely, Images6 (Research Centre for Medical Genetics, Moscow). The software determines the dot location, measures the nearest background signal, and calculates the integral dot intensity. Signals from several dots for the same sample were averaged, and the mean and standard error are calculated. The 8-oxodG content in a studied sample is calculated using the calibration curve equation. Relative standard error of the index 8-oxodG was 15 ± 5%.

### Peripheral blood lymphocyte isolation and culture

Peripheral blood lymphocytes (PBL) were isolated from heparinazed peripheral blood by Ficoll-Verographin density gradient centrifugation. The cells were washed twice in RPMI-1640 medium (ICN, USA) supplemented with 10% heat inactivated donor horse serum, 2 × 10^− 3^ M HEPES, 2 mM l-glutamine, 2.8 × 10^− 6^ M 2-mercaptoethanol, and 20 μg/mL gentamycin. The cells were cultivated in flat-bottomed 24-well plates (Costar, USA), which contained 10^6^ cells per well.

### Flow cytometry analyses

PBL subsets were evaluated with a flow cytofluorometer (СyFlow, Partek, Germany). Briefly, following isolation, the subsets were washed in PBS, split into tubes, and incubated for 30 min at 4 °C in the dark with fluorochrome-labeled monoclonal antibodies (phycoerythrin (PE)-conjugated anti-human CD3, fluorescein isothiocyanate (FITC)-conjugated anti-human CD4, FITC-conjugated anti-human CD8, FITC-conjugated anti-human CD14, FITC-conjugated anti-human CD19; eBioscience, San Diego, CA). For each analysis, 20,000 events were acquired and gated on CD4, CD8, or CD14 expression and side scatter properties. Samples were first run with single fluorochrome-stained preparation for color compensation.

Flow cytometry was also applied to measure levels of phosphorylated and non-phosphorylated forms of р65 protein (a subunit of NF-kB) in freshly isolated PBL and in PBL treated with oxidized DNA fragments. Primary anti-human р65 (‘Santa Cruz’, USA) and secondary (anti-mouse-FITC, SC-2010, ‘Santa Cruz’, USA) monoclonal antibodies were used according to the common protocol: the cells were washed with 1% albumin solution in PBS, fixed with 3.7% formaldehyde for 10 min at 37 °C, washed off, and permeabilized in 90% methanol at − 20 °C. Then, the cell suspension was incubated with primary antibodies (1 μg/mL) overnight at + 4 °C (1 μg/mL in PBS in the presence of 1% albumin) and, if necessary, with secondary antibodies for 1 h at room temperature in the dark and assayed with a flow cytofluorometer (СyFlow, Partek, Germany). Data on NF-kB content in the lymphocyte nuclei were obtained using a system for cell imaging ‘CyTell’ (‘General Electric Healthcare’, USA).

### Cytokine assays

EDTA plasma samples were analyzed for IL-1β, TNFα, IL-8, IL-17A, IL-10, and IFNγ (CYTOKINE; St.-Petersburg, Russia) using the enzyme-linked immunosorbent assay (ELISA) technique with commercially available kits in accordance with the manufacturer’s instructions.

### Measuring gene expression levels using real-time PCR

Expression levels of the genes *NFKB1*, *IL1В*, *IL8*, *IL6*, *ТВР*, and *GAPDH* were measured using real-time PCR. RNA was extracted from the cells using YellowSolve kits (Clonogen, Russia) or Trizol reagent (Invitrogen) as per manufacturer’s instructions (http://tools.lifetechnologies.com/content/sfs/ manuals/trizol reagent.pdf) with the subsequent phenol-chloroform extraction and precipitation with chloroform and isoamyl alcohol (49:1). RNA samples were treated with DNase without RNase activity (Silex, Russia) to remove the DNA contaminations. RNA concentrations were determined by using the Quant-iT RiboGreen dye RNA reagent (‘MoBiTec’, Germany) in a plate reader (EnSpire equipment, Finland) (*λ*_excit_ = 487 nm, *λ*_flu_ = 524 nm). The reverse transcription reaction was carried out using chemical reagents supplied by the Sileks company (Russia) according to the standard procedure. PCR was carried out using the corresponding primers (Syntol) and the intercalating dye SybrGreen at StepOnePlus instrument (‘Applied Byosystems’, USA). The used primers were as follows (written using the format (F; R)): *NFKB1* (5′-CAGATGGCCCATACCTTCAAAT-3′; 5′-CGGAAACGAAATCCTCTCTGTT-3′); *ТВР* (5′-GCCCGAAACGCCGAATAT-3′; 5′-CCGTGGTTCGTGGCTCTCT-3′); *GAPDH* (5′-GAAGGTGAAGGTCGGAGTC-3′; GAAGATGGTGATGGGATTTC-3′); *IL1B* (5′-GGTGTTCTCCATGTCCTTTGTA-3′; 5′-GCTGTAGAGTGGGCTTATCATC-3′); *IL8* (5′-ACTGAGAGTGATTGAGAGTGGAC-3′; 5′-AACCCTCTGCACCCAGTTTTC-3′); *TNFA* (5′-ATCAATCGGCCCGACTATCTC-3′; 5′-GCAATGATCCCAAAGTAGACCTG-3′).

The composition of the PCR reaction mix in a volume of 25 μL were the following: 2.5 μL of PCR buffer (700 mM/L Tris-HCl, pH 8.6; 166 mM/L ammonia sulfate, 35 mM/L MgCl_2_), 2 μL of 1.5 mM/L dNTP solution, and 1 μL of 30 picomol/L solution of each primer and cDNA. The conditions of PCR were chosen individually for each primer pair. The standard conditions for most primers were the following: after denaturation (95 °C, 4 min), 40 amplification cycles were conducted in the following mode: 94 °C for 20 s, 56 to 62 °С for 30 sec, 72 °С for 30 sec, and then 72 °С for 5 min. The PCR procedures were performed at StepOnePlus (Applied Biosystems, USA). PCR product lengths were checked by electrophoresis on a 1% agarose gel (if a better sensitivity was needed, a PAGE method was used). In addition, amplicon sequence identities for every primer pair were regularly verified by sequencing.

The expression levels of genes of interest were normalized to the expression levels of the respective standard gene (*TBP*, *GAPDH*). In experiments on cultivated lymphocytes, gene expression levels were measured in a series of independent tests on cells from different donors. Statistical analysis of the results was performed using a calibrating curve and after taking into account the PCR efficiency; the standard error was 2%.

### Preparation of oxidized DNA samples in vitro

The model oxidized DNA fragments (DNAoxy) for the experiments were prepared using combined treatment of selected genomic DNA sample with 300 mM Н_2_O_2_ and UV light (wavelength *λ* = 312 nm, 1.5 min, 25 °C). The modified DNA was precipitated with two ethanol volumes in the presence of 2 M ammonium acetate, washed twice with 75% ethanol, then dried, and dissolved in water. According to data of the ESI-MS/MS method, the content of 8-oxodG in model fragments was 1200 8-oxodG molecules per 10^6^ deoxynucleosides, or 1200AU (this value is equivalent to the actual level of 8-oxodG in the cfDNA samples under severe oxidative stress). It is assumed that the genomic DNA oxidation by H_2_O_2_ in vitro eliminates the effects of other possible factors influencing the cfDNA properties, such as changes in the methylation level or other different sequence contents [55].

### Statistical analysis

The statistical data analysis was conducted using MS Excel, Statistica 6.0, and StatGraph software. The null hypotheses of the absence of the difference between the compared samples were tested with the Mann–Whitney *U* test. Samples were deemed to be distinct at *p* < 0.05.

## Results

### cfDNA parameters and nuclease activity in blood plasma of ASD patients compared to a healthy control group

Table 1 represents the results of cfDNA quantification in plasma samples derived from ASD patients and healthy controls.

**Table 1:** Plasma biomarkers in patients with ASD and unaffected subjects

Parameters		Healthy controls (*n* = 27)	Patients with ASD				
*Detection limit*			Group I (*n* = 62)	р1*	Group II (*n* = 71)	р1*	р2**
cfDNA, ng/ml	*25 ng/ml*	389.4 ± 26 (151.8–732.6)	512.9 ± 197.8 (115.0–11338.0)	*0.014*	1151.2 ± 175.7 (78.7–4692.3)	*< 0.001*	*< 0.001*
Nuclease activity, U/ml	*1 U/ml*	8.6 ± 1.1 (3.3–29.2)	9.9 ± 1.0 (1.8–33.6)	*0.599*	10.4 ± 0.7 (4.4–30.7)	*0.089*	*0.271*
8-oxodG/10^6^ deoxynucleosides	*0.5/10*^*6*^*deoxynucleosides*	4.8 ± 0.9 (0.6–20.6)	3.3 ± 0.6 (0.5–20.6)	*0.107*	638.3 ± 313.2 (0.8–10478.9)	*< 0.001*	*< 0.001*

Group I, the patients with mild-to-moderate ASD; group II, the patients with severe ASD. Data are presented as mean ± SEM (minimum value**–**maximum value). The results were analyzed using Mann–Whitney *U* test. *p1, in comparison with healthy controls; **p2, in comparison with group I

As can be seen from the data of Table 1, cfDNA parameters have high variability. Nonetheless, a statistically significant increase in plasma cfDNA level in the groups of ASD patients compared with healthy controls has been observed. Thus, in group II (severe ASD), the median value of cfDNA concentration was approximately threefold higher than the same index in healthy controls (*р* < 0.0001). In the patients of group I (mild-to-moderate ASD), the augmentation of cfDNA concentration compared to normal baseline was less pronounced (by a factor of 1.3; *р* = 0.0136). It should be noted that cfDNA level in plasma of severe ASD patients significantly exceeded this parameter in the group of patients with mild-to-moderate ASD (*р* < 0.0001).

A study of nuclease activity in plasma samples from ASD patients and healthy children revealed no statistically significant differences. The ranges of endonuclease activity in all the groups were approximately the same and equal to 1.8–4.4 to 29.2–33.6 U/mL. There was a moderate tendency towards increase of nuclease activity in the plasma of group II compared in the healthy controls (10.4 vs 8.6 U/mL; *р* = 0.089). The findings suggest that the accumulation of cfDNA in the bloodstream of children with ASD may be partly associated with a reduced effectiveness of the cfDNA elimination system.

The assay of 8-oxodG content in cfDNA samples obtained from ASD cases and healthy controls revealed a significant elevation of this index in group II (see Table 1). The median level of 8-oxodG content in cfDNA samples from patients from group I was even lower than the index in healthy controls. A significant negative correlation between cfDNA concentration and 8-oxodG content has been found in ASD patients (Table S1 and S2). In the same time, we did not find correlations of CARS and SCQ scores with plasma cfDNA concentrations, nuclease activity, or 8-oxodG content (see Table S2).

### Plasma cytokine concentrations

Compared to healthy controls, patients with ASD demonstrated higher concentrations of pro-inflammatory cytokines IL-1β, IL-8, and IL-17A (Table 2). At the same time, the levels of anti-inflammatory cytokine IL-10 in the groups of ASD patients were 4–9 times lower than the respective values in the control group. Moreover, regardless of the disease severity, the ASD groups showed a moderate, but statistically significant decrease of IFNγ. It should be noted that mean values of plasma TNFα concentrations in ASD patients and healthy controls were below the detectable limit of ELISA kit we used (the data not shown). There were significant positive correlations between cfDNA concentration and plasma cytokine levels (IL-10, IL-17a, IFNγ) in ASD patient group (see Table S2). In the same time, we did not find correlations of CARS and SCQ scores with plasma cytokine concentrations (see Table S2).

**Table 2:** Plasma cytokine concentrations in patients with ASD and unaffected subjects

Plasma cytokines, pg/ml		Healthy controls		Patients with ASD			
	*Detection limit*		Group I	p1*	Group II	p1**	p2***
IL-1β	*3 pg/ml*	0.1 ± 0.1 (0.0*–17.3)	3.2 ± 1.6 (0.0–45.1)	*0.024*	5.3 ± 1.6 (0.0–64.8)	*0.005*	*0.525*
IL-8	*10 pg/ml*	726.8 ± 96.0 (171.4–5747.2)	2401.6 ± 387.2 (314.7–7938.3)	*0.005*	3966.0 ± 369.5 (238.0–8803.2)	*< 0.001*	*0.007*
IL-17A	*20 pg/ml*	34.2 ± 19.6 (12.3–54.0)	45.8 ± 20.1 (12.3–629.2)	*< 0.001*	69.9 ± 22.5 (14.4–1019.3)	*< 0.001*	*0.022*
IL-10	*5 pg/ml*	35.5 ± 7.1 (1.3–121.6)	4.0 ± 0.6 (1.3–18.0)	*< 0.001*	9.8 ± 3.9 (1.0–203.1)	*< 0.001*	*0.015*
IFNγ	*20 pg/ml*	72.4 ± 16.2 (28.3–175.2)	51.9 ± 29.8 (0.0–662.1)	*0.021*	65.621.4 (0.0–825.4)	*0.151*	*0.014*

Group I, the patients with mild-to-moderate ASD; group II, the patients with severe ASD. Data are presented as mean **±** SEM (minimum value**–**maximum value). * The data below the detectable limit were equated zero. The results were analyzed using nonparametric Mann–Whitney *U* test. **p1, in comparison with healthy controls; ***p2, in comparison with group I

### Gene expression of *NFκB1* and pro-inflammatory cytokines *TNFα*, *IL1β*, and *IL8* in PBL of patients with ASD and unaffected controls

**Fig. 1:**
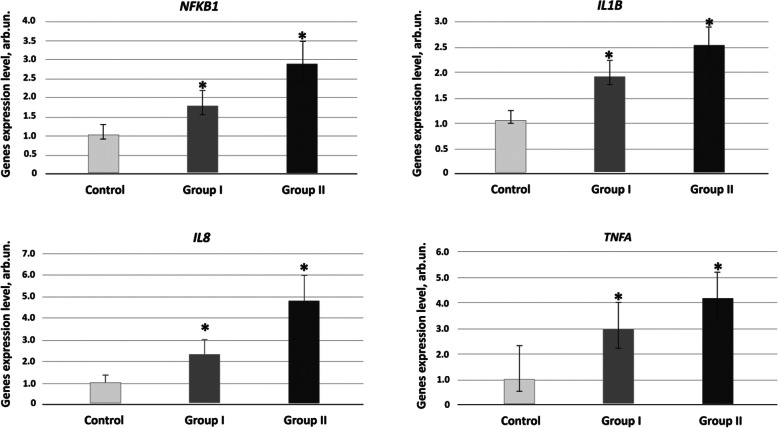
NF-κB/cytokine mRNA expression profiles of ASD patient and healthy control PBML. Transcript levels for NFκB, IL1β, IL8, and TNFα were evaluated by RT-qPCR analysis in PBL from control subjects and ASD patients. Each sample had been analyzed in triplicate. Samples were normalized according to ТВР and GAPDH expression. Results are presented as means ± SD. Group I, the patients with mild-to-moderate ASD; group II, the patients with severe ASD. *p value < 0.01 compared to healthy controls (Mann–Whitney *U* test)

The findings above are in line with the data obtained from studies of expression levels of the genes for *NFКB1* transcription factor and pro-inflammatory cytokines *TNFα*, *IL-1β*, and *IL8* in PBL of ASD patients and unaffected controls. As can be seen from the results presented in Fig. 1, PBL of ASD patients are characterized by heightened *NFКB1* gene expression. In group I, this index was by a factor of 1.5 to 2 higher than in the control group (*р* < 0.01); in group II, this index by a factor of 2.5 to 3 exceeded the respective parameter in the controls (*р* < 0.01).

After its activation, transcription factor NF-kB is translocated to the cell nucleus and launches the NF-kB signaling pathway that results in the start of expression of the target genes—a battery of pro-inflammatory cytokines. Studying the expression of genes for pro-inflammatory cytokines in peripheral blood mononuclear cells of mild-to-moderate ASD cases (group I) showed that the expression levels of *IL1β* and *IL8* were by a factor of 1.7 to 2.4 (both *р* < 0.01) higher, while the levels of *TNFα* were two- to fourfold higher (*р* < 0.01) compared to those of the control group. In group II of severe ASD patients, *IL1β* gene expression level was elevated by a factor of 2.5 to 3.5 (*р* < 0.01), and the expression of genes *IL8* and *TNFα* was elevated by a factor of 3.5 to 5.5 (*р* < 0.01).

Quantification of nuclear factor NFкB using flow cytometry revealed an increase in this parameter by 30–40% in group I, while group II showed an increase by a factor of 1.5 to 2 (Fig. S1)

As such, the data indicate that ASD patients have demonstrated increased cfDNA concentration in their circulation. cfDNA of patients with severe ASD has been characterized by a high abundance of oxidative modification. Additionally, ASD patients of both groups have shown elevated plasma cytokine (IL-1β, IL-8, IL-17A) levels, as well as heightened expression of genes for NFкB nuclear factor and genes for cytokines IL-1β, TNFα, and IL-8 compared to the control group.

### Model fragments of oxidized DNA alters transcriptional activity of genes for pro-inflammatory cytokines in vitro

The next stage of the study was involved the testing of a hypothesis that an accruing fraction of oxidized cfDNA in ASD patient’s blood plasma can activate a series of signaling cascades involved in inflammatory response. In particular, we explored the influence of DNAoxy (50 ng/ml) on the level of expression of the genes *NFКB1* and pro-inflammatory cytokines *TNFα*, *IL-1β*, and *IL-8* in PBL of patients with mild-to-moderate ASD (*n* = 18) and healthy controls (*n* = 11).

**Fig. 2:**
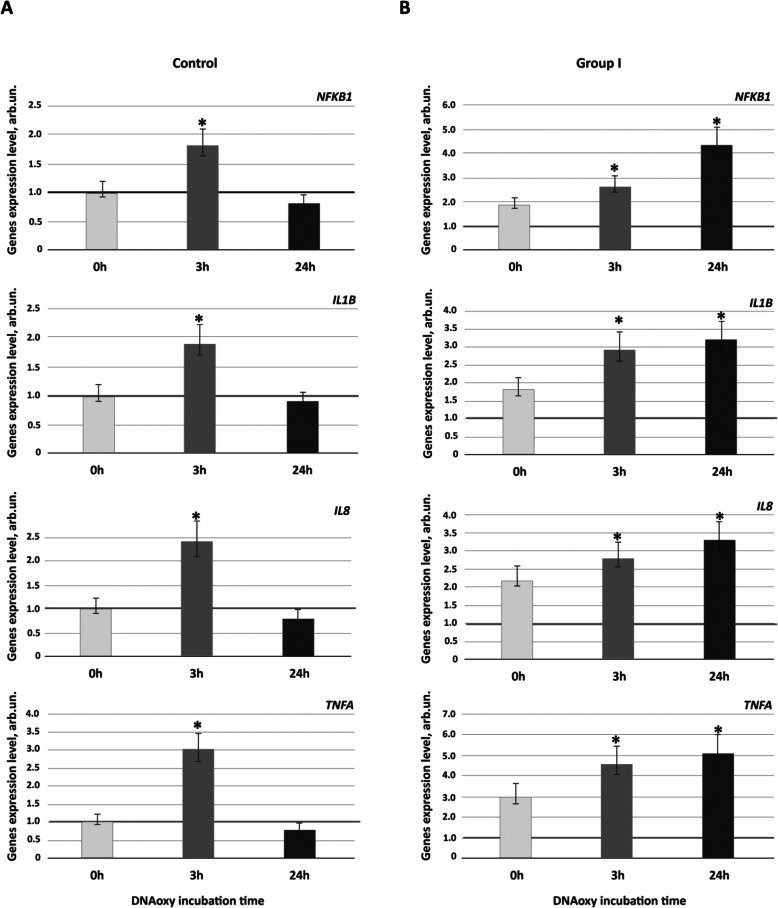
NF-κB/cytokine mRNA expression profiles of ASD patient and healthy control PBL treated with DNAoxy. PBL from control subjects and ASD patients were incubated with DNAoxy (50 ng/mL) for 3 and 24 h. Transcript levels for NF-κB, IL-1β, IL-8, and TNFα were evaluated by RT-qPCR analysis. Samples were normalized according to *ТВР* and *GAPDH* expression. The mRNA abundance is expressed relative to NF-κB/cytokine mRNA level in intact PBL of healthy controls (horizontal green line, 1.0 ± 0.3 arb.un). Data are shown as means ± SD. **a** Control, PBL from healthy children. **b** Group 1, PBL from patients with mild-to-moderate ASD; *p value < 0.01 compared to intact PBMC of healthy controls (Mann–Whitney *U* test)

It has been shown that NFκB/cytokine mRNA expression profiles of ASD patient PBL treated DNAoxy were significantly different from those of healthy controls (Fig. 2). Indeed, after 3 h incubation with DNAoxy, PBL of both healthy controls and patients with ASD have shown two- to fourfold elevation of *NFКB1* and pro-inflammatory cytokine (*TNFα*, *IL1β*, and *IL8*) gene expression (all *р* < 0.01). In 24 h after adding DNAoxy to the PBL cultures, the cells derived from healthy controls showed reduced expression of the genes mentioned above. While for ASD patients, PBL incubated with DNAoxy during 24 h displayed a further increase in *NFКB1* and pro-inflammatory cytokine (*TNFα*, *IL1β*, and *IL8*) gene expression (*р* < 0.01).

**Fig. 3:**
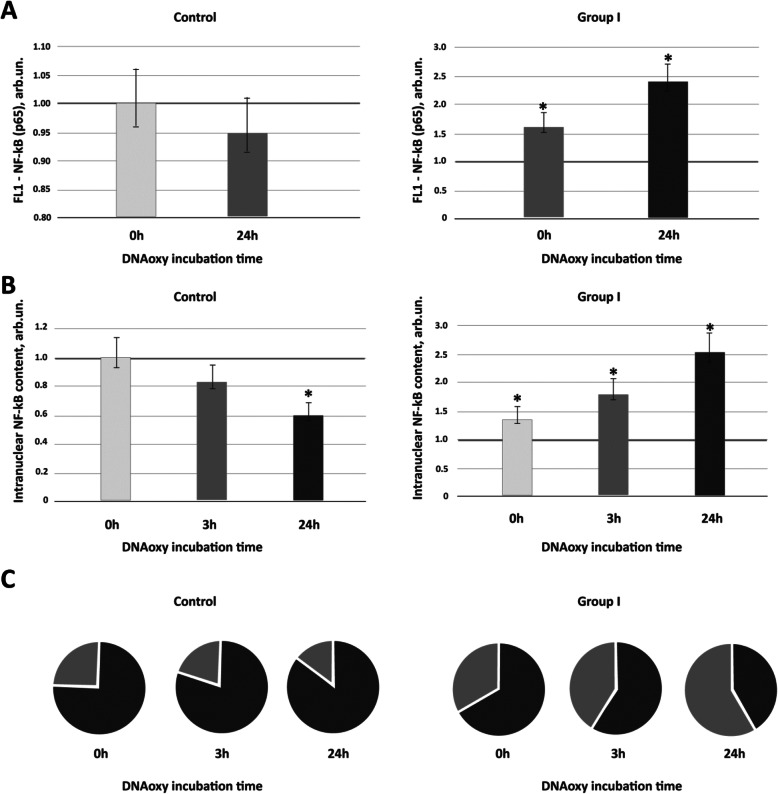
Total NF-κB (p65) expression and nuclear translocation of p65 as parameters of response to DNAoxy. PBL were cultured with DNAoxy (50 ng/ml) for various time durations up to 24 h. **a** Total NF-κB (p65) expression was quantified by flow cytometry. Expression level was measured as fold change of intensity relative to untreated zero time-point set in the control group (horizontal green line, 1.0 ± 0.3 arb.un.). For healthy controls, no change in expression level of p65 had been observed; for ASD patients, significant increase in p65 expression was found both at 0 h and 24 h time-points. Data are shown as means ± SD. **b** Relative cell number with intranuclear p65 location. PBL number with intranuclear p65 location were assessed by Cytell Cell Imaging System and expressed relative to untreated zero time-point set in the control group (horizontal green line, 1.0 ± 0.3 arb.un.). Data are shown as means ± SD. **c** Reads of the CyTell cell imaging system (GE Healthcare). The red sectors indicate the fraction of NF*κ*B-positive nuclei, while the blue sectors indicate the fractions of NF*κ*B-negative cell nuclei. The time of cultivation with DNAoxy (50 ng/ml) are indicated in the figure. Control, PBL of healthy controls; group 1, PBL of patients with mild-to-moderate ASD. *p value < 0.01 compared to untreated zero time-point set in the control group (Mann–Whitney *U* test

Similarly, flow cytometry analysis has shown that in 24 h after adding DNAoxy to the ASD patient PBL culture, expression level of NF-kB (p65) was fourfold higher than in PBL culture derived from healthy controls (*р* < 0.01; Fig. 3a).

Experiments using CyTell cell imaging system (GE Healthcare) have shown that 3 h after adding DNAoxy, the relative cell numbers with intranuclear NF-kB (p65) location in PBL cultures derived from ASD patients increased, while the relative cell numbers with cytoplasmic NF-kB (p65) location were reduced. After 24 h, the process of NF-kB translocation to the nuclei of ASD patient PBL was even more prominent (Fig. 3b, c). With regard to the PBL of healthy controls, there were no significant differences in the relative cell numbers with intranuclear NF-kB (p65) location before and after 3 h incubation with DNAoxy. However, 24 h after adding oxidized DNA fragments, the cell counts with nuclear NF-kB location in exposed control’s lymphocytes was significantly lower than in non-treated control’s cells.

## Discussion

ASDs are associated with a wide range of heterogeneous and, one would think, unrelated factors, such as toxins that penetrate maternal–fetal interface during pregnancy, environmental damaging impacts, vitamin D level at birth, child’s gender, and genetic factors. Risk factors for ASD development can be divided into two general categories: (1) inflammation-linked factors and (2) oxidative stress-linked factors [36]. We demonstrated previously that oxidative stress, both endogenous and induced by environmental damaging impacts, can induce oxidative cfDNA modifications [38, 39]. We surmised that the autistic blood may contain an increased amount of oxidized cfDNA, which is a stress-signaling factor capable of activating the chronic inflammatory process.

Our findings completely corroborate the hypothesis we put forward. We demonstrated that cfDNA content in patients with severe ASD substantially exceeded the contents in healthy controls and in patients with mild-to-moderate disease. At the same time, the endonuclease strength of ASD plasma samples was at a low level and did not differ from that in healthy controls. This data is evidence of a decline of the efficiency of cfDNA elimination system in patients with ASD and accumulation of cfDNA in blood circulation of children with autism. Due to the exhaustion of the antioxidant system in patients with severe ASD, circulating cfDNA undergoes oxidation which results in higher concentrations of 8-охоdG (see Table 1).

A significant negative correlation between cfDNA concentrations and 8-oxodG levels found in ASD patients (see Tables S1 and S2) is not an unexpected discrepancy. Similar results had been obtained in our previous investigations [56]. It is known that GC-rich genome sequences are accumulated in cfDNA of elderly healthy subjects and patients with chronic diseases [56]. A distinctive feature of GC-rich DNA fragments is their ability to be easily oxidized [38]. Under an acute process accompanied by the death of a large number of cells (stroke, heart attack, trauma, ionizing radiation), a large amount of DNA from dying cells enters the circulation during the first hours. In case of explosive increase in cfDNA concentration (high level of cell death), the content of GC-rich repeats and levels of oxidative modifications in cfDNA pool are relatively low [56–59]. When the acute process fades or becomes chronic, the level of cell death decreases. Concurrently, cfDNA is hydrolyzed by endonucleases and fragmented to small molecule fragments that are eliminated from the bloodstream. GC-rich DNA sequences are highly resistant to fragmentation and circulate as high molecular weight fragments that are eliminated more slowly. Accumulation of easily oxidized GC-rich sequences in cfDNA pool can lead to increase in the level of the oxidative modifications [56, 60–62].

Under conditions of excessive free radicals, blood cells demonstrate heightened levels of NFКB1 gene expression at the transcription and translation levels, followed by the launching of the NF-kB signaling pathway that, in turn, results in surplus production of pro-inflammatory cytokines and the emergence of aberrant inflammatory response in children with ASD.

It may be proposed that ASD progression can be mediated by signaling cascades that involve oxidized cell-free DNA fragments. Oxidized cfDNA, possibly via DNA sensors, activates the NF-kB-signaling pathway followed by a launch of an inflammatory process, which elevates the production of pro-inflammatory cytokines, including IL-8 and IL-17 (see Table 2). IL-8 and IL-17 are major factors that maintain aberrant neutrophilic inflammation. One of the consequences of excessive neutrophil activation is netosis, which is characterized by a release of genome DNA to the blood which, in turn, leads to the replenishment of the cfDNA pool [63].

We showed earlier that model fragments of oxidized DNA has the property of penetrating cell’s structures located close to the nuclear membrane, provoking activation of DNA sensors, and inducing a boost of gene expression of the NF-kB signaling pathway [41, 42]. We proposed that ASD patient PBL response to oxidized DNA treatment is markedly differed from that of healthy controls. Twenty-four hours after adding DNAoxy to PBL cultures, the cells derived from healthy controls showed reduced expression of *NFКB1* and pro-inflammatory cytokine (*TNFα*, *IL-1β*, and *IL-8*) gene expression, while ASD patients’ PBL incubated with DNAoxy for 24 h showed a further increase.

Our findings corroborate the assumption of a close link between oxidative stress and aberrant inflammatory response in patients with autism via oxidized cfDNA fragments. An important consequence of chronic oxidative stress is the permanent formation of single and double-strand DNA breaks, which activate PARP-1(poly (ADP-ribose) polymerase-1), as we showed earlier [64]. The overactivity of this enzyme, while followed by a depletion of ATP and aggravation of oxidative stress, also promotes formation of pro-inflammatory immunologic phenotype. Oxidized cfDNA fragments can perhaps boost the level of *NFКB1* expression via TLR8-receptors, resulting in launching NF-kB-signaling pathway that, in turn, results in the surplus production of pro-inflammatory cytokines and the emergence of aberrant inflammatory response in children with ASD.

## Limitations

The major limitation of our study is that it was not designed to identify the levels of GC-rich fragments in cfDNA samples of ASD patients. The negative correlation between cfDNA concentrations and 8-oxodG levels in plasma samples of ASD patients suggest accumulation of GC-reach fragments in their cfDNA pools (see above). Oxidized GC-rich fragments can penetrate cell membranes, accumulate in the cytoplasm, and interact with DNA sensors including TLR9, AIM2, and STING [56]. Undoubtedly, we will address to the investigation of GC-rich fragments in cfDNA pools of ASD patients in our future work.

## Conclusion

The results we obtained show evidence of altered characteristics of cfDNA in patients with ASD and, for the first time, suggest the significance of cfDNA as a regulator of signaling pathways in ASD. We demonstrated that cfDNA accruing in the blood stream of patients with ASDs and containing a high abundance of oxidative lesions can play an essential role in the regulation of gene expression and activation of pro-inflammatory signaling pathways in the PBL of ASD patients.

## Supplementary information

**Additional file 1: Figure S1.** NF-κB (р65) quantification in PBL of ASD patients and healthy controls. A – Representative histograms of cell NF-κB expression, evaluated by flow cytometry. B – Average of NF-κB expression in PBL of ASD patients and healthy controls. Data are presented as means ± SD. Group I – the patients with mild-to-moderate ASD; Group II – the patients with severe ASD. * - p < 0.01, compared to healthy controls (Mann-Whitney U test).

**Additional file 2: Table S1.** Spearman’s rank correlation between plasma cfDNA concentrations and 8-oxodG content in cfDNA samples.

**Additional file 3: Table S2.** Spearman’s rank correlation (R) between clinical and laboratory parameters in ASD patients.

## Data Availability

All data generated or analyzed during this study are included in this published article.

## Abbreviations

ASD: Autism spectrum disorder
cfDNA: Cell-free DNA
DNAoxy: Model oxidized DNA fragments
IFN: Interferon
IL: Interleukin
*NFКB*, NF-kB: Nuclear factor kappa-light-chain-enhancer of activated B cells
PARP-1: Poly (ADP-ribose) polymerase-1
PBL: Peripheral blood lymphocytes
TLR: Toll-like receptor
TNF: Tumor necrosis factor
