# Supplementary material for: Oxidized cell-free DNA as a stress-signaling factor activating the chronic inflammatory process in patients with autism spectrum disorders
Source: J Neuroinflammation. 2020 Jul 16;17:212. doi: 10.1186/s12974-020-01881-7 (PMC7364812; doi:10.1186/s12974-020-01881-7)
Supplement: Supplementary file 1 — Additional file 1: Figure S1. NF-κB (р65) quantification in PBL of ASD patients and healthy controls. A – Representative histograms of cell NF-κB expression, evaluated by flow cytometry. B – Average of NF-κB expression in PBL of ASD patients and healthy controls. Data are presented as means ± SD. Group I – the patients with mild-to-moderate ASD; Group II – the patients with severe ASD. * - p < 0.01, compared to healthy controls (Mann-Whitney U test). [file 12974_2020_1881_MOESM1_ESM.docx]

**Supplementary information**


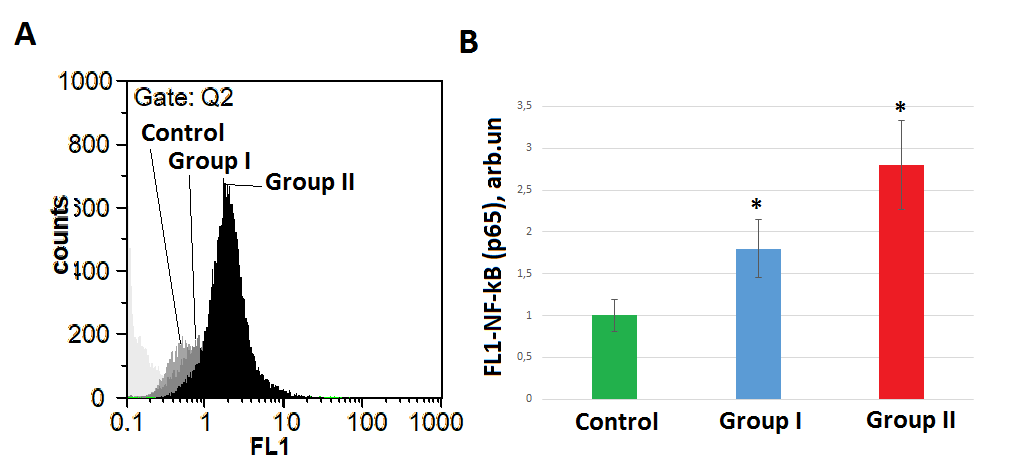


**Fig.S1.** NF-κB (р65) quantification in PBL of ASD patients and healthy controls. A – Representative histograms of cell NF-κB expression, evaluated by flow cytometry. B – Average of NF-κB expression in PBL of ASD patients and healthy controls. Data are presented as means ± SD. Group I – the patients with mild-to-moderate ASD; Group II – the patients with severe ASD. * - p < 0.01, compared to healthy controls (Mann-Whitney U test).
