# Supplementary material for: Oxidized cell-free DNA as a stress-signaling factor activating the chronic inflammatory process in patients with autism spectrum disorders
Source: J Neuroinflammation. 2020 Jul 16;17:212. doi: 10.1186/s12974-020-01881-7 (PMC7364812; doi:10.1186/s12974-020-01881-7)
Supplement: Supplementary file 2 — Additional file 2: Table S1. Spearman’s rank correlation between plasma cfDNA concentrations and 8-oxodG content in cfDNA samples. [file 12974_2020_1881_MOESM2_ESM.docx]

**Supplementary information**

Table S1. Spearman’s rank correlation between plasma cfDNA concentrations and 8-oxodG content in cfDNA samples

|  | N | R | p |
| --- | --- | --- | --- |
| Healthy controls | 27 | -0.278 | 0.160 |
| Group I (mild-to-moderate ASD) | 62 | -0.569 | **0.002** |
| Group II (severe ASD) | 71 | -0.569 | **<0.001** |
