# Supplementary material for: Oxidized cell-free DNA as a stress-signaling factor activating the chronic inflammatory process in patients with autism spectrum disorders
Source: J Neuroinflammation. 2020 Jul 16;17:212. doi: 10.1186/s12974-020-01881-7 (PMC7364812; doi:10.1186/s12974-020-01881-7)
Supplement: Supplementary file 3 — Additional file 3: Table S2. Spearman’s rank correlation (R) between clinical and laboratory parameters in ASD patients. [file 12974_2020_1881_MOESM3_ESM.docx]

**Supplementary information**

Table S2. Spearman’s rank correlation (R) between clinical and laboratory parameters in ASD patients

|  | **CARS** | **SCQ** | **cfDNA** | **Nuclease activity** | **8-oxodG** | **ИЛ-1β** | **ИЛ-8** | **ИЛ-10** | **ИЛ-17а** |
| --- | --- | --- | --- | --- | --- | --- | --- | --- | --- |
| **SCQ** | **0,71*** |  |  |  |  |  |  |  |  |
| **cfDNA** | 0,09 | 0,10 |  |  |  |  |  |  |  |
| **Nuclease activity** | -0,16 | -0,25 | -0,26 |  |  |  |  |  |  |
| **8-oxodG** | -0,16 | -0,05 | -**0,57*** | -0,01 |  |  |  |  |  |
| **ИЛ-1β** | 0,10 | -0,04 | 0,14 | 0,01 | -0,04 |  |  |  |  |
| **ИЛ-8** | -0,21 | -0,27 | 0,27 | 0,06 | 0,02 | 0,16 |  |  |  |
| **ИЛ-10** | 0,00 | -0,07 | **0,42*** | -0,18 | 0,20 | 0,22 | **0,36*** |  |  |
| **ИЛ-17а** | 0,13 | -0,04 | **0,41*** | 0,12 | 0,05 | 0,24 | **0,49*** | **0,44*** |  |
| **ИФγ** | 0,15 | -0,07 | **0,46*** | 0,10 | 0,23 | **0,31*** | **0,48*** | **0,53*** | **0,78*** |

CARS - Childhood Autism Rating Scale (score); SCQ - Communication Questionnaire (score); ***** – p<0.01.
